# Integrated luminometer for the determination of trace metals in seawater using fluorescence, phosphorescence and chemiluminescence detection

**DOI:** 10.1155/S1463924602000081

**Published:** 2002

**Authors:** P. J. Worsfold, E. P. Achterberg, A. R. Bowie, V. Cannizzaro, S. Charles, J. M. Costa, F. Dubois, R. Pereiro, B. San Vicente, A. Sanz-Medel, R. Vandeloise, E. Vander Donckt, P. Wollast, S. Yunus

**Affiliations:** 1 Department of Environmental Sciences Plymouth Environmental Research Centre University of Plymouth Plymouth PL4 8AA UK; 2 Department of Physical and Analytical Chemistry University of Oviedo c/Julián Clavería 8 Oviedo E-33006 Spain; 3 Chimie Organique Physique Université Libre de Bruxelles 50 Av. F. D. Roosevelt Brussels 1050 Belgium; 4 Laboratoire de Traitement des Eaux et Pollution Université Libre de Bruxelles 50 Av. F. D. Roosevelt Brussels 1050 Belgium

## Abstract

The paper describes an integrated luminometer able to perform fluorescence (FL), room temperature phosphorescence (RTP) and chemiluminescence (CL) measurements on seawater samples. The technical details of the instrumentation are presented together with flow injection (FI) manifolds for the determination of cadmium and zinc (by FL), lead (RTP) and cobalt (CL). The analytical figures of merit are given for each manifold and results are presented for the determination of the four trace metals in seawater reference materials (NASS-5, SLEW-2) and Scheldt estuarine water samples.


Journal of Automated Methods & Management in Chemistry
Vol. 24, No. 2 (March–April 2002) pp. 41–47

Taylor & Francis

Taylor & Francis Group

### Integrated luminometer for the determination of trace metals in seawater using fluorescence, phosphorescence and chemiluminescence detection

**P. J. Worsfold[1*], E. P. Achterberg[1], A. R. Bowie[1], V. Cannizzaro[1], S. Charles[3], J. M. Costa[2], F. Dubois[3], R. Pereiro[2], B. San Vicente[2], A. Sanz-Medel[2], R. Vandeloise[3], E. Vander Donckt[3], P. Wollast[4] and S. Yunus[3]**

*[1]Department of Environmental Sciences, Plymouth Environmental Research Centre, University of Plymouth, Plymouth PL4 8AA, UK [2]Department of Physical and Analytical Chemistry, University of Oviedo, c/Julián Clavería 8, E-33006 Oviedo, Spain [3]Chimie Organique Physique and [4]Laboratoire de Traitement des Eaux et Pollution, Université Libre de Bruxelles, 50 Av. F. D. Roosevelt, 1050 Brussels, Belgium*

*The paper describes an integrated luminometer able to perform fluorescence (FL), room temperature phosphorescence (RTP) and chemiluminescence (CL) measurements on seawater samples. The technical details of the instrumentation are presented together with flow injection (FI) manifolds for the determination of cadmium and zinc (by FL), lead (RTP) and cobalt (CL). The analytical figures of merit are given for each manifold and results are presented for the determination of the four trace metals in seawater reference materials (NASS-5, SLEW-2) and Scheldt estuarine water samples.*

### Introduction

It is important to determine trace metal concentrations in marine (and freshwater) environments in order to monitor compliance with legislation and ensure good water quality [1]. There are various techniques available, but the family of luminescence techniques—fluorescence (FL), room temperature phosphorescence (RTP) and chemiluminescence (CL) [2]—offers the potential for highly selective and sensitive determinations [3], providing that the appropriate reagents can be found and the methods optimized. The challenge of determining trace metals in seawater environments is compounded by the potential interference from matrix constituents, particularly the major ions.

This paper reports on the development of a combined luminometer that can be readily switched between the various luminescence modes to provide optimized methods for the determination of a range of trace metals. Samples and reagents are presented to one of the photomultiplier-based detectors by means of automated flow-injection manifolds controlled by the instrument software. Where necessary, matrix interferences are removed by means of in-line microcolumns containing chelating or ion-exchange resins [4, 5]. The accuracy of the various manifolds has been determined by analysis of certified seawater reference materials. The ability to perform sequential determinations of different analytes in the same sample using the three different modes has been effectively demonstrated using Scheldt estuarine water samples. Cadmium, zinc, lead and cobalt were determined using FL (Cd, Zn), RTP (Pb) and CL (Co) and the results were validated against standard laboratory methods.

*To whom correspondence should be addressed. e-mail: pworsfold@plymouth.ac.uk

### Experimental

### *Reagents*

Analytical reagent-grade chemicals were used throughout and high-purity water (Milli-Q, Millipore, Watford, UK) was used for the preparation of all solutions unless otherwise stated. For the determination of Cd and Zn using FL, anthylazamacrocycle **1** (9-(1′,4′,7′,10′,13′,16′-hexa-azacyclooctadecyl)methyl-anthracene ) was synthesized in the laboratory in Brussels according to literature procedures [6] (for further details, see below). Optimum reagent conditions included 0.5 μM anthylazamacrocycle **1** in NaOH (Micro Select, Fluka, Buchs, Switzerland), resulting in a reaction pH 9 for Zn and 13 for Cd determinations. Dowex 1X2-400 (Supelco, Bornem, Belgium) anion-exchange resin was cleaned before use with 1 M HCl (37% Trace Select, Fluka) for 30 min, and then with Milli-Q water for 1 h. The resin was packed in a 10-ml column (Omnifit, Cambridge, UK). Between samples, the Dowex column was cleaned by pumping a solution of 1 M NaCl/0.01 M HCl over the resin (NaCl, > 99%, ACS reagent, Aldrich, St Louis, MO, USA). About 9.5 ml NaCl (5 M) and 312 μl HCl (concentrated) per 50-ml sample was added to samples and standards prior to injection onto the column. The column was eluted using a solution of 0.75 M NaCl/0.065 M HCl.

For the determination of Pb(II) using RTP, 8-hydroxy-7-quinoline sulphonic acid (Fluka) was used without further purification. Aqueous solutions of the reagent were prepared by dissolving the appropriate amount of the solid reagent in Milli-Q water. Dowex 1X2-200 anion exchange resin (Sigma, Munich, Germany) was cleaned thoroughly before use with 2 M HCl to remove trace metal impurities, then with Milli-Q water and finally with ethanol to displace air from the pores of the resin and to remove residual monomers and solvents. The resin was placed into the RTP flow-cell (volume about 25 μl; see below for further details).

Reagents for the FI–CL determination of Co(II) included pyrogallol (1,2,3-trihydroxybenzene, ACS grade, Aldrich, Gillingham, UK), methanol (HPLC-grade, Rathburn, Walkerburn, UK), CTAB (cetyl-trimethylammonium bromide, Microselect 99%, Fluka), sodium hydroxide and hydrogen peroxide (both Analar, Merck, Poole, UK). The optimum reagent concentrations were 50 mM pyrogallol, 25 mM CTAB, 1 M hydrogen peroxide, 20% v/v methanol and pH 10.35 (0.15 M sodium hydroxide). A 0.1 M ammonium acetate buffer (Merck) was used for on-line pH buffering (pH 5.1) of the sample before Co preconcentration on an 8-hydroxyquinoline (8-HQ) microcolumn. The chelating microcolumn (Perspex casing) was packed with 8-HQ immobilized on a commercial hydrophilic vinyl co-polymer (Toyopearl TSK gel, HW 75F, 32–63 micron fine, TosoHaas, Stuttgart, Germany) according to [5]. The 8-HQ column contained about 0.05 ml resin and had a lifetime of at least 6 months. A 0.05 M HCl (quartz-distilled) solution was used as the microcolumn elution solution. The 8-HQ column was cleaned daily by rinsing for 10 min with 0.01 M HCl.

Standard solutions for calibration of the various luminescence techniques were prepared daily from appropriate stock solutions (Spectrosol, Merck). Standard solutions for the different elements were prepared separately. Certified reference materials were obtained from the National Research Council of Canada.

Estuarine water samples were collected from the Scheldt estuary near Antwerp, Belgium, on an ebb tide (25 July 2000). Salinity was measured *in situ* using a homemade salinometer. Samples were filtered immediately after collection through an acid-cleaned (0.1 M HCl, 24 h) 0.45 µm polycarbonate membrane filter (Sartorius) in a field-laboratory on a pontoon in the estuary. Samples were then acidified to pH 1.5 with 200 µl HNO3 (65% Suprapur, Merck Eurolab, Ontenay Sous Bois, France) per 100 ml sample.

*Instrumentation*

The luminometer was a modified version of a commercial instrument (FL/FS 900 from Edinburgh Analytical Instruments) capable of monitoring fluorescence (FL) and room temperature phosphorescence (RTP) decay curves after pulsed excitation, FL and RTP steady-state signals and transient chemiluminescence (CL) emission. A block diagram of the instrumentation is shown in figure 1. Emission was monitored at 90° to the excitation beam for FL and RTP. A flipping mirror was incorporated to select the appropriate light source in front of the excitation monochromator and a special cell compartment for CL measurements was added. Excitation wavelengths (200–750 nm) were selected by a 300-mm focal length grating (G318 HOµ25) blazed at 250 nm. Emission wavelengths (200–900 nm) were selected by a second monochromator equipped with a grating (G318HOµ5) blazed at 500 nm. A Hamamatsu R1527 PMT was used to measure light intensities in the photon counting mode.

The excitation source used for steady-state FL was a continuous 450 W Xe-arc (Xe 900, 230–2000 nm). The light source used to record time-resolved fluorescence emission was an nF 900-nanosecond flashlamp filled with hydrogen. The source emitted approximately $10^8$ photons per pulse in the wavelength range 110–850 nm with a FWHM of 0.8 ns. Its power output was > 5 W at 100 Hz; its frequency could be adjusted between 1 and 100 Hz and its FWHM was in the range 1.5–3.0 µs. The pulses were viewed by a 9661B Electron Tubes PMT by the time-correlated single photon counting (TCSPC) technique. A µF 900 ns flashlamp served as an excitation source for monitoring RTP spectra and decay profiles. The delay and gate times were 0.05 ms and the excitation and emission slits were set at 10 and 20 nm, respectively. CL signals were measured with an R 6095 Hamamatsu PMT.

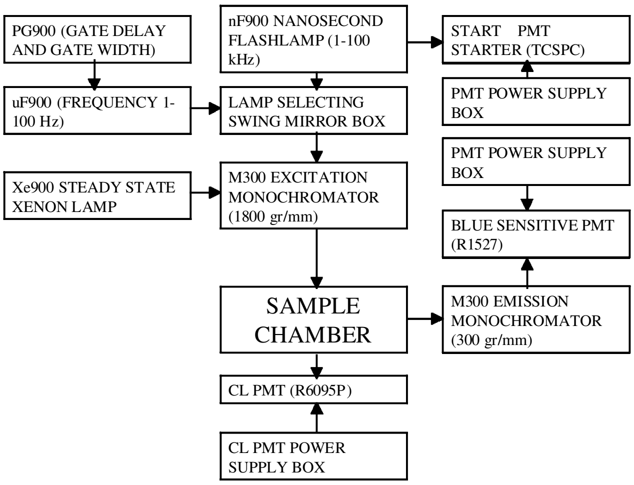

*Figure 1. Block diagram of the integrated luminometer.*

A PC controlled the luminometer. Data acquisition and analysis were performed by the FS900 CDT Edinburgh Instruments Software for CL and steady-state FL and RTP emissions. Data acquisition and analysis in the TCSPC mode were performed with the FL 900 CDT and FLA 900 level 2 software packages.

*Fluorescence FI manifold for cadmium and zinc*

The FI manifold shown in figure 2 was used for the sequential determination of Cd and Zn in a sample. The analytical methodologies used the same reagents, eluents and elution procedures for the two metals, with the exception of the use of a reaction pH 9 for Zn analysis and pH 13 for Cd analysis. The manifolds for Cd and Zn were identical, with three channels, one for each metal and one for the reagent solution. Zinc and Cd were therefore determined separately with a changeover of reagent solution. The solutions were pumped and injected on the Dowex 1X2-400 microcolumns and through the fluorescence cell via a three-way peristaltic pump (Gilson Minipuls 3, Villiers le Bel, France). A mixing 'T'-piece (Cole Parmer, Vernon Hills, IL, USA), PTFE tubing (0.8 mm i.d.) and peristaltic pump tubing (Elkay, Shrewsbury, MA, USA) were used to connect the reservoirs, the three-way valves and the flow-through cell (450 µl, Hellma). The two metal lines were alternately connected to the waste and to the fluorescence flow-through cell in such a way that during the preconcentration step, the eluted fraction was discarded

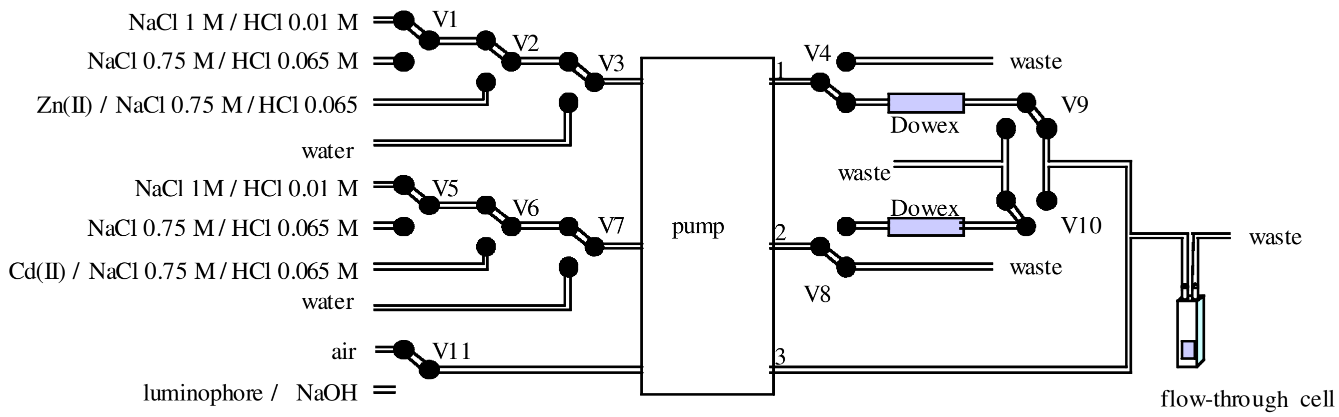

*Figure 2. FL manifold for Cd and Zn.*

while during the wash step, the metals were passed through the fluorescence cell.

Flow rates for lines 1 and 2 (figure 2) were 2.1 and 0.9 ml min$^{-1}$, respectively. The resin was washed for 1 min using eluent 1. The samples or standards were injected for 5 min (line 2). The metals were eluted using eluent 2 (2 min), eluent 1 (3.5 min) and Milli-Q water, successively. The eluted metals was then mixed with the reagent, and after on-line complexation the metal chelate was passed through the flow-through cell where its fluorescence was measured. The fluorescent measurement took about 200 s. A complete analytical cycle was performed within 17 min using a 5-min preconcentration.

Ten three-way PTFE solenoid switching valves V1–10 were used to direct the different solutions sequentially through the flow-through cell. The valves were controlled via the parallel port of the PC using a Cool-Drive™ 24-V DC command interface. The sequences were fully automated using an in-house computer program written in Visual Basic. The graphic interface software (figure 3), constructed in an Excel environment, had three distinctive components. The first was a table showing the different sequences of elution. It distinguished between the introduction step, the main step (which could be repeated several times) and the end step. Each step was assigned a different code (1, 2 or 3) for the introduction step, the main step and the end step respectively. The second part was a diagram of the manifold showing the currently active part of the manifold, e.g. valves, peristaltic pump. Clicking on the RUN button started a new run. The third part was an information panel that gave the times for the start and finish of each step and the nature of the current operation, e.g. preconcentration, elution.

The control device for the operation of the valves was interfaced with the PC (figure 4, item 1) via the parallel port (Centronics). Adaptation of the levels and protection of the parallel port were done by means of an octal

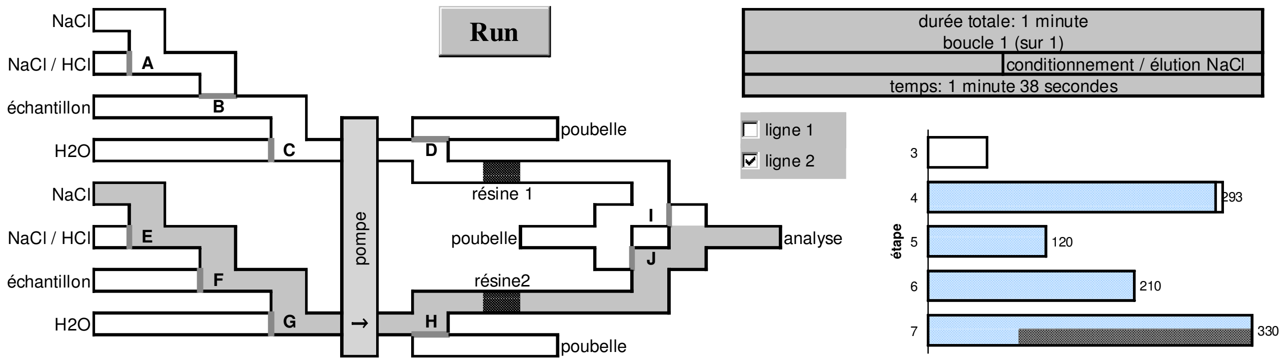

| Etape | 1 | 2 | 3 | 4 | 5 | 6 | 7 | 8 | 9 | 10 | Durée | Proc. | Code1 | Code2 | Code3 | Code4 |
|---|---|---|---|---|---|---|---|---|---|---|---|---|---|---|---|---|
| 1 | 0 | 1 | 0 | 1 | 0 | 0 | 0 | 0 | 1 | 0 | 10 | 1 | 85 | 21 | 128 | 0 |
| 2 | 0 | 0 | 1 | 1 | 0 | 0 | 0 | 0 | 1 | 0 | 20 | 1 | 86 | 22 | 128 | 0 |
| 3 | 0 | 0 | 0 | 0 | 0 | 0 | 0 | 0 | 1 | 0 | 60 | 2 | 80 | 16 | 128 | 0 |
| 4 | 0 | 1 | 0 | 0 | 0 | 0 | 0 | 0 | 1 | 0 | 300 | 2 | 81 | 17 | 128 | 0 |
| 5 | 1 | 0 | 0 | 0 | 0 | 0 | 0 | 0 | 1 | 0 | 120 | 2 | 88 | 24 | 128 | 0 |
| 6 | 0 | 0 | 0 | 0 | 0 | 0 | 0 | 0 | 1 | 0 | 210 | 2 | 80 | 16 | 128 | 0 |
| 7 | 0 | 0 | 1 | 0 | 0 | 0 | 0 | 0 | 1 | 0 | 330 | 2 | 82 | 18 | 128 | 0 |
| 8 | 0 | 0 | 1 | 0 | 0 | 0 | 0 | 0 | 1 | 0 | 30 | 3 | 82 | 18 | 128 | 0 |

*Figure 3. Graphic interface for the control of the integrated luminometer.*

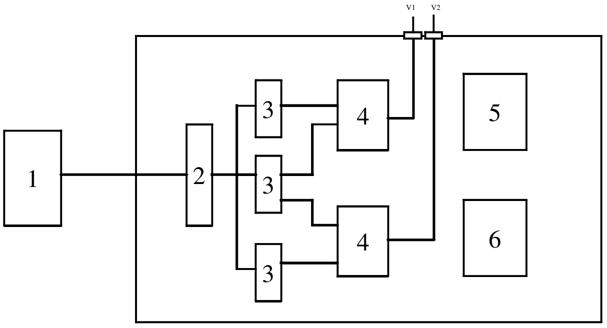

*Figure 4. Control device for operation of the valves. 1 = PC parallel port; 2 = 74HCT541; 3 = 74HCT75; 4 = valve drivers 225D5X24; 5 = power supply 5 V DC; 6 = power supply 24 V DC.*

buffer and line drivers with a three-state output integrated circuit (74HCT541) (figure 4, part 2). Since the number of valves that needed to be controlled (10) was larger than the number of data lines (eight) of the parallel port, demultiplexing and storage of the valve positions was necessary. These two functions were fulfilled by means of three integrated 74HCT75 circuits (Dual 2-Bit Bistable Transparent Latch) (figure 4, part 3). Two valve drivers (CoolDrive 225D5X24 Valve Drivers, NResearch, Inc.) were used to power the valves (figure 4, part 4). To minimize interference problems, two independent power supplies were used, 24 V DC for the valves and 5 V DC for the integrated circuits.

### *Room temperature phosphorescence FI manifold for lead*

The FI manifold for the determination of Pb(II) is shown in figure 5. A four-channel peristaltic pump (Gilson Minipuls 2) was used to generate the flowing streams. Rotary valves (Omnifit 1106) were used for sample and reagent introduction (valves A and B) and for the elution of the retained species. An Omnifit 2401 mixing T-piece, PTFE tubing (0.8 mm i.d.) and fittings were used to connect the flow-through cell, the rotary valves and the carrier solution reservoirs. Samples (2 ml) were injected via valve A and chelating reagent solution (2 ml) through valve B into the flow system. Both solutions, at a flow rate of 1.4 ml min$^{-1}$, were mixed in a T-piece and passed through the 0.5-m reaction coil to ensure chelate formation. After on-line reaction, the anionic Pb complex passed into the detector flow cell (Hellma Model 176.52-QS, 25 µl, Müllheim, Baden, Germany), placed inside the sample compartment of the luminometer, and was retained on a packing of the Dowex 1X2-200. At the bottom of the flow cell a small piece of nylon net was placed to prevent particle displacement by the carrier stream. The Dowex resin was loaded with the aid of a syringe and the exit end of the flow cell was kept free. Then, the cell was connected to the flow system and 10 min was allowed for the particles to settle. To ensure that the compound retained by the packing solid material was in the light path, the resin level was maintained 1 mm below the cell window.

The RTP intensity was then measured at an excitation wavelength of 395 nm and an emission wavelength of 595 nm. After measurement, 500 µl 6 M HCl was injected via valve C (to strip the chelate retained on the solid phase) before proceeding with the next sample injection.

### *Chemiluminescence FI manifold for cobalt*

The FI manifold used for the CL determination of Co(II) is shown in figure 6. Two peristaltic pumps (Gilson Minipuls 3) were used to deliver the sample and buffer, Milli-Q water, eluent and reagents. All manifold tubing was PTFE (0.75 mm i.d., Fisher) except for the peristaltic pump tubing, which was Tygon (Elkay). The flow rates for the various sample and reagent flows are presented in figure 6. A six-port PTFE rotary injection valve (Rheodyne, model 5020) was used for sample introduction. The flow cell was a quartz glass spiral (1.1 mm i.d., 130 µl internal volume) positioned in front of a mirror in the sealed housing. The reagent flow created a background CL emission of 0.15 mV with a peak-to-peak noise of 0.1 mV. The operating procedure for the FI–CL Co manifold was as follows: all PTFE flow lines, fittings and connectors of the FL manifold were initially cleaned with 0.5 M quartz-distilled HCl and UHP water for at least 2 h. For the determination of Co(II) in seawater, sample was buffered in-line to pH 5.1 with ammonium acetate (0.1 M) and loaded onto the 8-HQ chelating resin column for 60 s at 1.2 ml min$^{-1}$. Milli-Q water was then passed through the column for 30 s to remove the major seawater cations and anions. The injection valve was switched to the elute position for 60 s and 0.05 M HCl was passed through the column in the reverse direction at 1.2 ml min$^{-1}$ to elute the Co(II). The eluent stream then

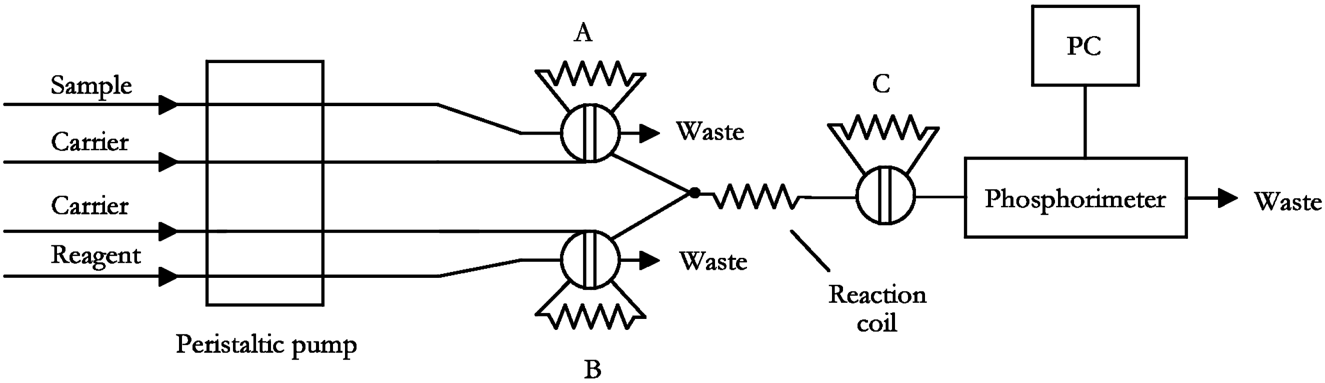

*Figure 5. RTP manifold for Pb.*

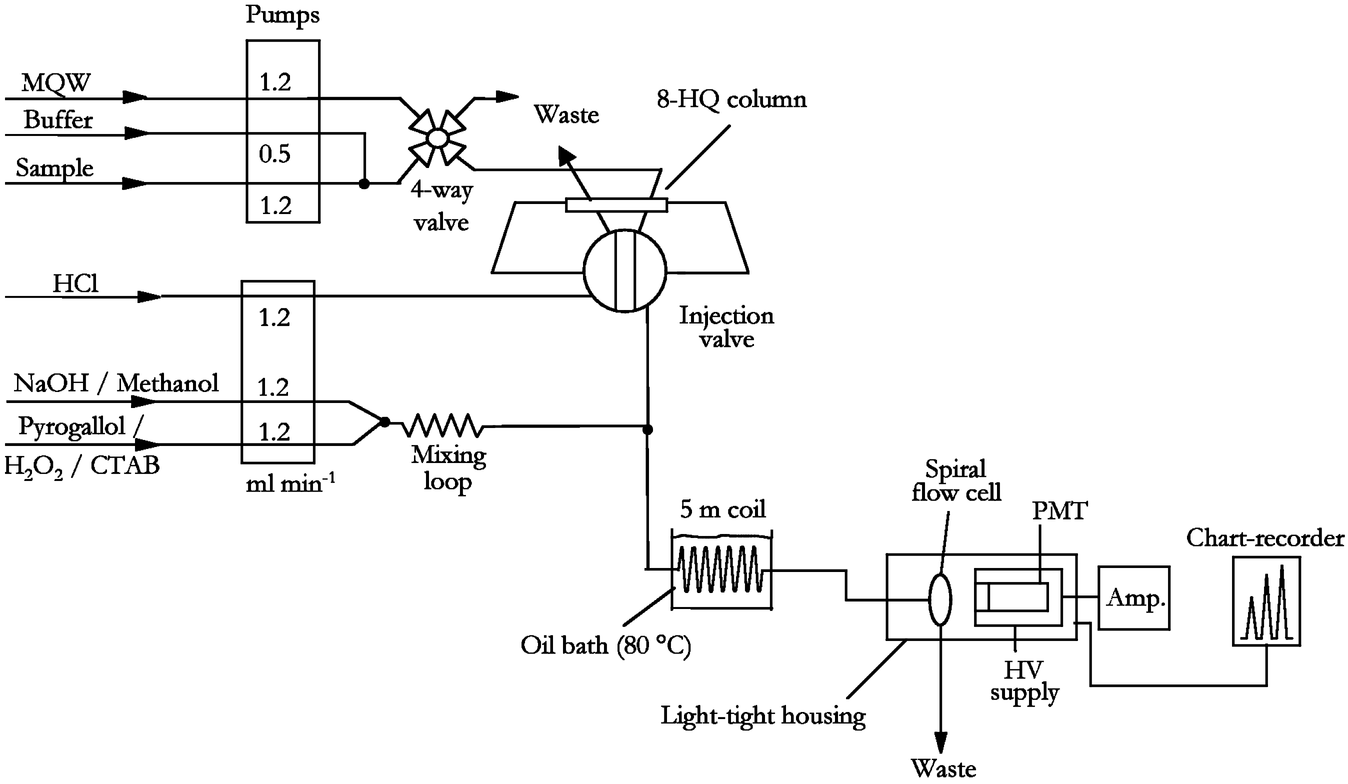

*Figure 6. CL manifold for Co.*

merged with the reagent solutions and passed through a 5-m reaction coil (immersed in a heated oil bath at 80°C) to the flow cell. The injection valve was then returned to the load position and washed with Milli-Q water for 30 s to remove residual HCl before starting the next load sequence.

### Results and discussion

### *Fluorescence determination of cadmium and zinc*

The method used to determine Cd and Zn was based on the fluorescence modification resulting from the binding of these metals to a polyazacrown covalently linked to a luminophore via one methylene group. In this work, the anthylazamacrocycle **1** (figure 7) was used [6, 7]. In aqueous alkaline conditions, the fluorescence yield of **1** was very small. This was ascribed to an intramolecular quenching by electron transfer from the amines to the excited fluorophore. When chelated to non-quenching metal ions such as Cd or Zn, the amine lone pairs become involved in bonding and cannot donate an electron to the excited state of the anthryl group. Consequently, chelation-enhanced fluorescence and a bathochromic shift of the spectra are observed. Elements including Cu(II), Hg(II) and Pb(II) interfere with the FL determination of Cd and Zn. The use of the Dowex 1X2-400 column not only resulted in a preconcentration of Cd and Zn on the column, but also allowed metals separation to prevent fluorescence quenching by other trace metals.

*Cadmium.* For Cd(II) standards and blanks prepared in Milli-Q water and preconcentrated for 5 min, the fluorescence response was linear over the range 35–2000 pM ($r^2 = 0.993$) and the limit of detection was 35 pM (3 s, $n = 5$). The relative standard deviation (SD) was 10% with a signal-to-noise ratio = 30 for a 200-pM Cd(II) standard. Variation of the preconcentration time from 5 to 20 min for a 500-pM Cd solution also gave a linear response ($r^2 = 0.998$), demonstrating that this was a good variable for altering the linear range to suit particular environmental conditions. The accuracy of the FI–FL technique for Cd was determined using marine-certified water reference materials (SLEW-2, NASS-5). A small baseline drift was observed during the analysis and corrected by a linear adjustment of the baseline before and after the elution peak. The results for SLEW-2 and NASS-5 obtained after this linear correction are given in

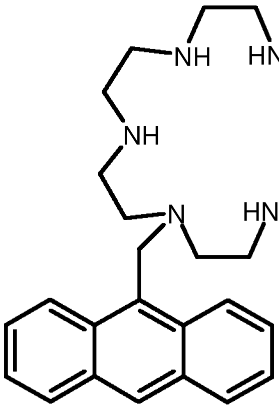

*Figure 7. Structure of the anthylazamacrocycle* **1** *used for the determination of Cd and Zn.*

table 1 and show good agreement with certified values [8].

*Zinc*. The fluorescence response was linear over the range 4–100 nM ($r^2 = 0.985$) for a 5-min preconcentration and the limit of detection was 4 nM (3 s, $n = 5$). The relative SD was 8% for a 50-nM Zn(II) standard. Variation of the preconcentration time from 30 s to 5 min gave a linear response ($r^2 = 0.980$) for a 100-nM Zn(II) standard and showed that preconcentration time could be reduced when determining Zn in highly contaminated samples. The high detection limit for Zn compared with that obtained for Cd can be explained by a higher background signal due to the residual fluorescence of the reagent at pH 9 and higher external contamination. The choice of CRMs to determine the accuracy of the method was therefore restricted to SLEW-2 and a coastal seawater sample from the Irish Sea. The data obtained for SLEW-2 and for the Irish Sea are shown in table 2 and they are in good agreement with certified values. Cd in SLEW-2 and the Irish Sea was 50–100 times less than Zn and therefore it did not interfere with the Zn determination.

### *Room temperature phosphorescence determination of lead*

The RTP method for the determination of Pb in seawater was based on the formation in a FI manifold (figure 5) of a Pb chelate with 8-hydroxy-7-quinolinesulphonic acid [9], which was then on-line immobilized on the anionic exchange resin Dowex 1X2-200. A flow rate of 1.4 ml min$^{-1}$, sample injection loop of 2 ml and a reaction coil of 500 µl ensured the complete formation of the metal chelate. Once the metal chelate was retained onto the Dowex resin and the RTP measurements were taken, the chelate was removed from the resin by 500 µl 6 M HCl. The maximum sampling frequency that can be achieved with the proposed optosensing method for Pb analysis was 12 samples h$^{-1}$. RTP measurements of the metal chelate were performed in the absence of oxygen (solutions contained $3 \times 10^{-3}$ M sodium sulphite). A delay time of 0.05 ms was used in all RTP measurements to ensure that any background fluorescence had ceased before RTP measurement. When using shorter delay times (i.e. 0.01–0.04 ms), residual fluorescence from the complex and the solid support itself was observed. The excitation spectrum of the complex showed intense absorption bands at 395 nm, and in the absence of oxygen, the RTP emission spectrum showed a maximum at 595 nm. The limit of detection was 0.5 nM, the relative SD was ±3% ($n = 4$) for a 0.5-µM Pb(II) standard solution. Moreover, a good regression coefficient for the FI-RTP calibration graph was obtained ($r = 0.9989$) over the range 0.5–10 000 nM.

A detailed study of potential interferences on the RTP determination of Pb(II) was performed. The presence of other heavy metals such as Zn, Cd and Co at concentrations several times higher than observed in seawater did not affect Pb(II) determination. Therefore, it was possible to determine Pb(II) using RTP in seawater in the presence of Cd, Zn, Pb and Co.

### *Chemiluminescence determination of cobalt*

This FI-CL method uses the oxidation of pyrogallol in the presence of methanol and cetyltrimethylammonium bromide, with a basic solution of hydrogen peroxide as the oxidant [9, 10]. A detection limit of 5 pM (3 s of the baseline noise) was achieved using the FI-CL manifold shown in figure 6, with a linear range ($r^2$ consistently $> 0.999$) of 0.5–850 pM. This shows that the method was suitable for the determination of Co(II) in coastal and open ocean waters. One sequence of triplicate injections of sample and three standard additions with a 60-s preconcentration (sample load) took 40 min. In interference studies [10], the only element showing any detectable interference with the Co FI-CL method was Ag(I), at concentrations 50 times the typical Ag concentration in open ocean seawater. Because the Ag concentration in seawater is typically 0.5–35 pM, it is therefore unlikely that it would interfere in the analysis. The accuracy of the method was determined by analysing three marine CRMs and an Irish Seawater sample and the results (table 3) show good agreement with the certified values (for the CRMs) and with a cathodic stripping voltammetry (CSV) method (for the Irish seawater).

### *Scheldt study*

The Scheldt is a perturbed estuarine system situated on the border between Belgium and The Netherlands. The River Scheldt originates in France and receives high

*Table 1. Cadmium concentrations in the CRMs SLEW-2 and NASS-5.*

| CRM | FI–FL (pM) | Certified value (pM) |
|---|---|---|
| SLEW-2 | 190 ± 34 | 170 ± 20 |
| NASS-5 | 213 ± 18 | 200 ± 30 |

Numbers are the mean ±2 s ($n = 4$).

*Table 2. Zinc concentrations in the CRM SLEW-2 and an Irish Seawater sample.*

| CRM | FI–FL (pM) | Certified value (pM) |
|---|---|---|
| SLEW-2 | 15.0 ± 2.5 | 16.8 ± 2.1 |
| SLEW-2 + 50 nM Zn(II) | 65 ± 7 | 67 |
| Irish seawater | 30 ± 6 | 27–29 |

Numbers are the mean ± 2 s.

*Table 3. Cobalt concentrations in the CRMs NASS-4, CASS-3 and SLEW-2 and an Irish Sea sample.*

| Sample | FI–CL (nM) | Certified value (nM) | CSV (nM) |
|---|---|---|---|
| NASS-4 | 0.16 ± 0.01 | 0.15 ± 0.02 | – |
| CASS-3 | 0.60 ± 0.09 | 0.68 ± 0.11 | – |
| SLEW-2 | 0.93 ± 0.13 | 0.87 ± 0.21 | – |
| Irish seawater | 0.35 ± 0.02 | – | 0.34 ± 0.01 |

Numbers are the mean ± 2 s ($n = 4$).

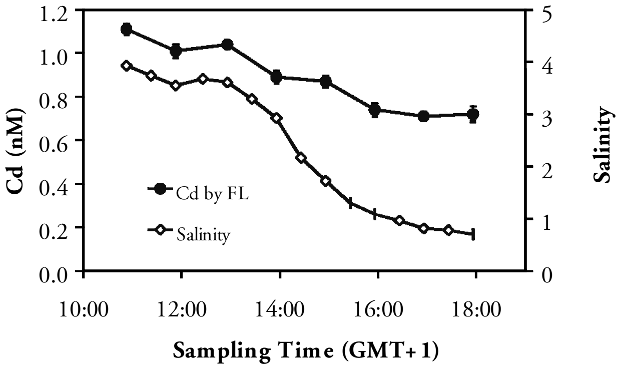

*Figure 8. Time series for dissolved Cd in the Scheldt with FL analysis.*

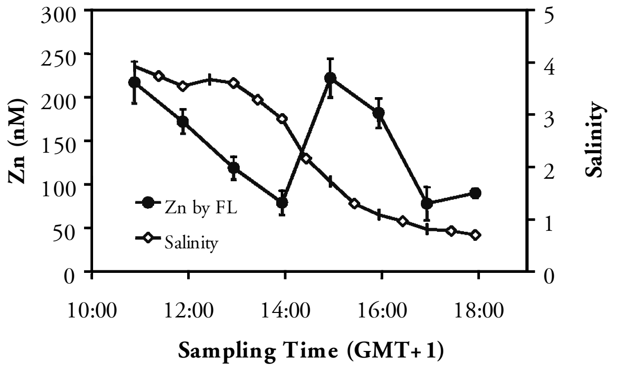

*Figure 9. Time series of dissolved zinc in the Scheldt with FL analysis.*

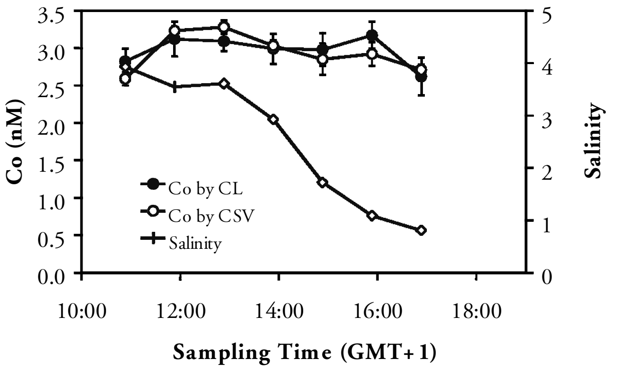

*Figure 10. Time series of dissolved Co in the Scheldt with CL and comparative CSV analyses.*

levels of waste water inputs. Samples were collected during an outgoing tide in the summer of 2000 as described above, and dissolved trace metal concentrations were determined following sample filtration at the University of Brussels, using the integrated luminometer. The time series data for dissolved Cd, Zn and Co are shown in figures 8–10 respectively. The Cd-salinity relationship was linear ($r^2 = 0.95$) and was assigned to the desorption of Cd from suspended solid matters due to the increasing concentration of chloride at high tide during the mixing of freshwater with seawater with the formation of dissolved Cd chloride complexes. Owing to the low concentrations of dissolved Pb present in the water samples, a preconcentration step before the analysis was necessary. The preconcentration was carried out

*Table 4. Dissolved Pb(II) in Scheldt samples determined using FI–RTP and ICP–MS.*

| Sample time (GMT + 1 h) | FI-RTP ($\mu g\,l^{-1}$) | ICP–MS ($\mu g\,l^{-1}$) |
|---|---|---|
| 13:53 | 1.2 | 0.9 |
| 14:53 | 1.3 | 1.2 |
| 15:53 | n.d. | n.d. |

by passing 500 ml of the water samples on-line through a microcolumn packed with 8-hydroxyquinoline (8-HQ) immobilized on a commercial hydrophilic vinyl co-polymer (Toyopearl TSK gel). After preconcentration, the Pb was eluted from the column by injecting 2 ml HCl (0.5 M), and this solution was subsequently injected in the FI manifold. The data for dissolved Pb are shown in table 4. Dissolved Pb and Co were also determined using ICP-MS and CSV respectively and the results showed good agreement with the data from the luminometer.

### Conclusions

The three different luminescence techniques (FL, RTP, CL) can be combined in a single instrument and flow injection manifolds can be used to pretreat and deliver samples to the relevant detector. Microcolumns are effective for removing interfering seawater matrix ions. The sensitivity is suitable for the determination of a range of trace metals (Cd, Zn, Pb, Co) in estuarine and coastal (and open ocean) waters with good accuracy. Supporting technical data can be found at the MEMOSEA website [3].

### Acknowledgements

The authors thank the EU for funding under the MAST Programme (Grant No. MAS3-CT97-0143, MEMOSEA).

### References

1. Worsfold, P. J., Achterberg, E. P., Bowie, A. R., Sandford, R. C. and Mantoura, R. F. C., 2000, in M. S. Varney (ed.), *Chemical Sensors in Oceanography* (Amsterdam: Gordon & Breech), 71.
2. Fletcher, P., Andrew, K. N., Calokerinos, A. C., Forbes, S. and Worsfold, P. J., 2001, *Luminescence*, **16**, 1.
3. http://www.ulb.ac.be/sciences/cop/MAST-III/main.htm
4. Nickson, R. A., Hill, S. J. and Worsfold, P. J. 1995, *Analytical Proceedings*, **32**, 387.
5. Landing, W. M., Haraldsson, C. and Paxeus, N., 1986, *Analytical Chemistry*, **58**, 3031.
6. Akkaya, E. U., Huston, M. E. and Czarnik, A. W., 1990, *Journal of the American Chemistry Society*, **112**, 3590.
7. Huston, M. E., Engleman, C. and Czarnik, A. W., 1990, *Journal of the American Chemistry Society*, **112**, 7054.
8. Charles, S., Yunus, S., Dubois, F. and Vander Donckt, E., 2001, *Analytica Chimica Acta*, **440**, 37.
9. Segura-Carretero, A., Costa-Fernandez, J. M., Pereiro, R. and Sanz-Medel, A., 1999, *Analytica Chimica Acta*, **395**, 1.
10. Cannizzaro, V., Bowie, A. R., Sax, A., Achterberg, E. P. and Worsfold, P. J., 2000, *Analyst*, **125**, 51.

